# AI-Based Enhancing of xBn MWIR Thermal Camera Performance at 180 Kelvin

**DOI:** 10.3390/s25103200

**Published:** 2025-05-19

**Authors:** Michael Zadok, Zeev Zalevsky, Benjamin Milgrom

**Affiliations:** 1 Faculty of Engineering, Institute for Nanotechnology and Advanced Materials, Bar-Ilan University, Ramat-Gan 5290002, Israel; zadokmi1@biu.ac.il; 2 School of Electrical Engineering, Jerusalem College of Technology, P.O. Box 16031, Jerusalem 9372115, Israel; milgromb@gmail.com

**Keywords:** xBn MWIR camera, 180 Kelvin operation, ESRGAN, high operating temperature (HOT), cost-effective thermal imaging, image quality assessment, SWaP

## Abstract

Thermal imaging technology has revolutionized various fields, but current high operating temperature (HOT) mid-wave infrared (MWIR) cameras, particularly those based on xBn detectors, face limitations in size and cost due to the need for cooling to 150 Kelvin. This study explores the potential of extending the operating temperature of these cameras to 180 Kelvin, leveraging advanced AI algorithms to mitigate the increased thermal noise expected at higher temperatures. This research investigates the feasibility and effectiveness of this approach for remote sensing applications, combining experimental data with cutting-edge image enhancement techniques like Enhanced Super-Resolution Generative Adversarial Networks (ESRGAN). The findings demonstrate the potential of 180 Kelvin operation for xBn MWIR cameras, particularly in daylight conditions, paving the way for a new generation of more affordable and compact thermal imaging systems.

## 1. Introduction

Thermal imaging has emerged as a powerful tool in remote sensing, providing valuable information about surface temperatures and thermal patterns. However, the sensitivity of thermal cameras is often limited by thermal noise, which can significantly degrade image quality.

Commercially available thermal cameras offer a diverse range of spatial resolutions, typically spanning from 160 × 120 pixels to 1280 × 1024 pixels [1] and even up to 2560 × 2048 pixels for new cameras [2], reflecting the complex interplay between achieving the desired image detail and optimizing Size, Weight, and Power (SWaP) characteristics [1]. This resolution dictates the level of detail that can be discerned in the thermal image. However, regardless of the inherent resolution of the camera, the quality of thermal images is often compromised by thermal noise. Thermal noise, an inherent characteristic of all electronic systems operating above absolute zero, arises from the random thermal motion of charge carriers within the camera sensor. This noise manifests random fluctuations in the detected signal, introducing graininess and reducing the clarity of the thermal image. There are several studies about the performance of HCT (HgCdTe) MWIR cameras that operate at higher temperatures up to 217 Kelvin [3,4] that allow for a reduction in camera size, weight, and price. However, there is no available research about the performance of xBn MWIR cameras that operate above 150 Kelvin.

xBn detectors, specifically InAsSb/AlSbAs XBn detectors, offer several advantages compared with HCT camera. For example: XBn detectors exhibit diffusion-limited dark currents, a nearly 40% weight and power reduction, and a better mean time to failure (MTTF) [5]. Therefore, it is important to check the performance of those types of cameras as well.

Traditionally, researchers have relied on various image processing techniques to mitigate the impact of thermal noise and enhance the quality of thermal images. Image enhancement encompasses a wide range of approaches aimed at improving the visual appeal and information content of an image, including techniques to sharpen details, adjust contrast, and reduce noise [6]. While these techniques can be effective to a certain extent, they often struggle to fully compensate for the inherent limitations posed by thermal noise, especially in low-light conditions or when imaging objects with subtle temperature variations. Traditional methods often rely on multiple images or prior knowledge, which may not always be available or practical. Furthermore, many algorithms struggle with distortions like saturation, a challenge also present in thermal imaging when temperature ranges exceed sensor capabilities [7].

The emergence of artificial intelligence (AI), particularly deep learning algorithms, has revolutionized the field of image processing, offering new and powerful tools for image enhancement and super-resolution. Super-resolution, a specific class of image enhancement, focuses on increasing the resolution of an image beyond its inherent limits, essentially recovering finer details that were lost during the image acquisition process. Early super-resolution (SR) models, such as Super-Resolution Generative Adversarial Network (SRGAN) [8] and ESRGAN [9], relied on bicubic kernel interpolation to downsample HR images during training, creating synthetic low-resolution (LR) images to learn the mapping between LR and SR image pairs. However, bicubic kernel interpolation, while computationally simple, only partially simulates the complexity of real-world image degradation, which often involves a diverse combination of degradations like blurring, noise, and compression artifacts. This limitation led to the development of blind SR models, designed to handle LR images with unknown and complex degradation processes [10].

Real-ESRGAN [11], a prominent example of a blind SR model, utilizes the HDM (High-order Deterioration Model) to more effectively simulate real-world image degradation during training. The HDM goes beyond simple interpolation methods, incorporating a combination of blurring, downsampling, noise addition, and compression to create a more realistic representation of real-world LR images [12]. This comprehensive approach enables Real-ESRGAN to learn more robust mapping between LR and HR image pairs, leading to improved reconstruction quality for real-world images.

Despite its advantages, Real-ESRGAN still faces challenges. Reconstructed images can sometimes appear overly smooth, resulting in a loss of fine texture details and, consequently, a performance that is inferior to classical models like SRGAN and ESRGAN in certain scenarios [12]. This over-smoothing is likely a consequence of the model prioritizing the removal of noise and artifacts, sometimes at the expense of fine textural details. Moreover, while Real-ESRGAN-reconstructed images might exhibit good visual quality, they can differ significantly from the original HR image, particularly in terms of fine textures and subtle details. This deviation, while not always visually apparent, raises concerns about the fidelity of the reconstruction and its suitability for applications where the accurate representation of fine details is critical.

A key advantage of ESRGAN is its ability to address the unique challenges of sensor models, including limited training data, diverse geographical features, and complex atmospheric conditions. This is achieved through training on paired low- and high-resolution remote sensing images and the potential use of data augmentation, demonstrating ESRGAN’s capacity to mitigate data limitations in remote sensing applications of deep learning [13].

Operating a thermal camera at 180 Kelvin, instead of the conventional 150 Kelvin, presents both opportunities and challenges. On the one hand, operating at a higher temperature could potentially lead to increased thermal noise, further degrading image quality. On the other hand, this approach allows for the exploration of a new operational regime for thermal cameras, potentially offering benefits in terms of cost, power consumption, or sensor sensitivity. Investigating the feasibility and benefits of operating thermal cameras at this higher temperature, while effectively mitigating the increased thermal noise, constitutes a novel and potentially impactful research direction.

This article aims to demonstrate the feasibility and effectiveness of operating an xBn MWIR thermal camera at 180 Kelvin for remote sensing applications, leveraging advanced AI algorithms like ESRGAN to compensate for the increased thermal noise. By combining this novel operational approach with cutting-edge image enhancement techniques, this research seeks to pave the way for a new generation of thermal imaging systems capable of delivering high-quality images even in challenging conditions.

By extending the operational range to 180 Kelvin, the price and size of a camera can be reduced significantly as the power of the cooling system can increase up to 20% according to the heat transfer formula:

(1) Q=U×A×ΔT

where

*Q* = heat transfer rate (W);
*U* = overall heat transfer coefficient (W/m^2^K);
*A* = surface area of the heat exchanger (m^2^);
*ΔT* = temperature difference between the hot and cold sides (K).

Therefore, the cooling power at 150 Kelvin is 0.01 × 150 K = 1.5 W, and for 180 Kelvin the cooling power = 0.01 × 120 K = 1.2 W. Therefore, the size reduction factor is 1.2 W/1.5 W = 0.8 or 20%. Usually, for high-end products, a reduction of 20% in performance can give a decrease of 50% percent in price and size, which can affect the entire design of the camera and reduce the price significantly. One can decide not to reduce the cooler volume but to enlarge the camera matrix. HOT cameras present design possibilities that are essential in many types of system such as airborne applications like MAWS (Missile Approach Warning System) and EO-DAS (Electro-Optical Distributed Aperture Systems) [14,15,16].

The primary objective of this study is to investigate the theoretical and experimental feasibility of operating xBn MWIR thermal cameras at 180 Kelvin. In this article, we focus on investigating the camera performance at various operating temperatures and several AI algorithms.

The subsequent sections of this article will detail the experimental methodology, data analysis, and results of this research. The objective is to showcase the effectiveness of this approach in improving the quality of thermal images acquired at 180 Kelvin, ultimately contributing to the advancement of thermal imaging technology for remote sensing applications.

## 2. Materials and Methods

For our experiments, we use an xBn MWIR camera of SCD company (Leshem Industrial, Caesarea, Israel), with 10 μm pixel pitch, 640 × 512 FPA, frame rate of 30 Hz, Nyquist resolution of 50 lp/mm, and A/D resolution of 13 bits. The camera had the ability to operate at different temperatures https://www.scd.co.il/products/blackbird-640-sparrow/ (accessed on 15 May 2025).

**Figure 1:**
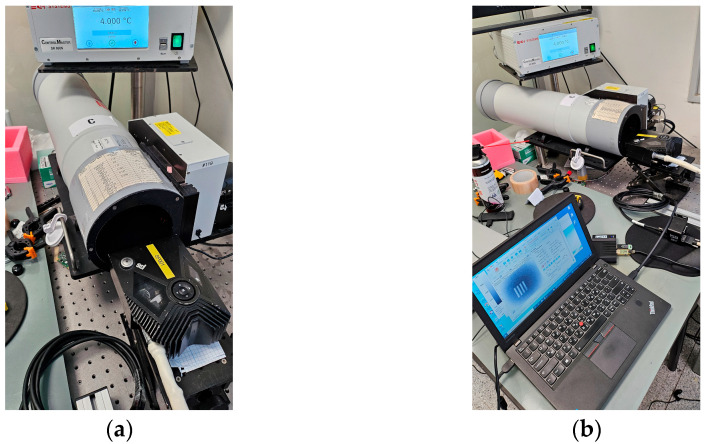
Setup of the experiment: SCD camera, collimator, laptop, (**a**) we can observe that ΔT=4 °C, (**b**) represents how the raw data are seen on the laptop during the experiment’s setup.

In our initial experiment, we used a USAF 1951 target positioned within a CI-Systems ilet5 collimator (Dallas, TX, USA), which was set to a temperature difference (ΔT) of 4 degrees. The camera was placed in front of the collimator, and initial testing was performed at 150 Kelvin, followed by further testing at 180 Kelvin. The setup of the experiment is shown in Figure 1.

**Figure 2:**
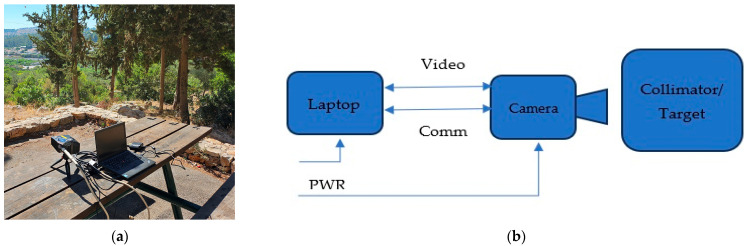
(**a**) Setup of the outdoor experiments: SCD camera, laptop, batteries; (**b**) schematic diagram of the setup.

The second experiment was conducted outdoors during daylight hours with pictures taken at a distance of 400 m at 150 and 180 Kelvin. The third experiment was conducted outdoors during the evening (in low light) with pictures taken at a distance of 400 m at 150 and 180 Kelvin. The fourth experiment was conducted outdoors during daylight hours with pictures taken at a distance of 800 m at 150 and 180 Kelvin. The fifth experiment was an open view test during the day with pictures taken at a distance of 1500 m at 150 and 180 Kelvin. All experiments were conducted in a warm environment, with ambient temperatures ranging from 23 to 30 degrees Celsius. Figure 2 illustrates the test setup for the outdoor experiments in (a), and a schematic diagram of the test set.

**Figure 3:**
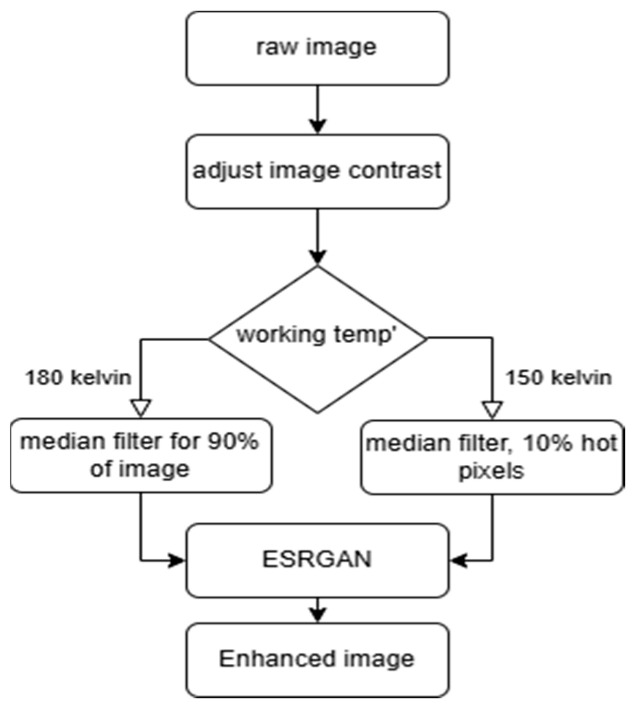
Enhancement flowchart.

The experimental raw data were enhanced through a sequence of general algorithms: (1). Image intensity was adjusted. (2). Hot pixels were removed using a median filter on adjacent pixels. This step involved temperature-specific thresholding: at 150 Kelvin, the filter activated for values above 0.9 (targeting extreme outliers), whereas at 180 Kelvin, a lower threshold of 0.1 was used, applying the filter more extensively due to increased noise. (3). The ESRGAN algorithm, not specifically trained for our data, was then applied. The flowchart outlining this entire process is shown in Figure 3.

In order to show the improvement of the images, we used a 2 step quality comparison: First, we compared the difference between the original image and the enhanced image at 150 Kelvin and at 180 Kelvin. The comparison was made using the NIQE (Naturalness Image Quality Evaluator) [17], a no-reference image quality score, because we did not have a reference image to compare with. The NIQE algorithm uses the comparison between the natural MVG (Multivariate Gaussian Model) and the distorted image’s MVG model. The formula used for the comparison is as follows:

(2) $$NIQE\left(v_{1},v_{2},\Sigma_{1},\Sigma_{2}\right)=\sqrt{{\left(v_{1}-v_{2}\right)}^{T}{\left(\frac{\Sigma_{1}-\Sigma_{2}}{2}\right)}^{-1}\left(v_{1}-v_{2}\right)}$$

where $v_{1},v_{2}$ and $\Sigma_{1},\Sigma_{2}$ are the mean vectors and covariance matrices, respectively, of the natural MVG model and the distorted image’s MVG model.

The second step involved comparing the enhanced images (at 150 and 180 Kelvin) with their original counterparts (at 150 and 180 Kelvin) to quantify the improvement achieved by the enhancement process. For this comparison, we employed several established image quality assessment methods [18,19,20,21,22,23,24,25], including Mean Squared Error (MSE), Peak Signal-to-Noise Ratio (PSNR), and the Universal Image Quality Index (UIQI). MSE calculates the average of the squared differences between the pixel values of the original and distorted images, using the following formula:

(3) $$MSE\left(I,K\right)=\frac{1}{mn}\sum_{i=1}^{n}\sum_{j=1}^{m}{\left[I\left(i,j\right)-K\left(i,j\right)\right]}^{2}$$

where *I*, *K* are the reference image and distorted image.

The units of MSE are “pixel value units squared”.

PSNR calculates the ratio of the maximum possible power of a signal to the power of the corrupting noise. A higher PSNR value indicates greater clarity in the distorted image. The PSNR is computed using the following formula as follows:

(4) $$PSNR\left(I,K\right)=10log10\left(\frac{R^{2}}{MSE(I,K)}\right)$$

where *R* is the maximum value (pixel) of input image.

MSE and PSNR only calculate absolute errors. They do not consider factors like luminance masking and contrast masking, which are aspects of human visual perception. Hence, they can sometimes be mismatched with perceived visual quality. The UIQI separates the comparison between the original and distorted images into three comparisons, namely: 1. Luminance, 2. Contrast, 3. Structural. These comparisons are calculated as follows:

(5) $$l\left(x,y\right)=\frac{2\mu_{x}\mu_{y}}{2\mu_{x}^{2}+\mu_{y}^{2}}$$

(6) $$c\left(x,y\right)=\frac{2\sigma_{x}\sigma_{y}}{\sigma_{x}^{2}+\sigma_{y}^{2}}$$

(7) $$s\left(x,y\right)=\frac{2\sigma_{xy}}{\sigma_{x}+\sigma_{y}}$$

where $\mu_{x},{\;\mu }_{y}$ are the mean values of original and distorted images, $\sigma_{x},\;\;\sigma_{y}$ are the standard deviation of original and distorted images, and $\sigma_{xy}$ is the covariance of both images. Based on the above three comparison equations, the formula used for the UIQI computation is as follows:

(8) $$UIQI\left(x,y\right)=l\left(x,y\right)c\left(x,y\right)s\left(x,y\right)\;$$

## 3. Results

In this section, we will show the images that were collected in the experiments, the enhancement that was made, and the improvements both at 150 Kelvin and 180 Kelvin.

### 3.1. First Experiment

**Figure 4:**
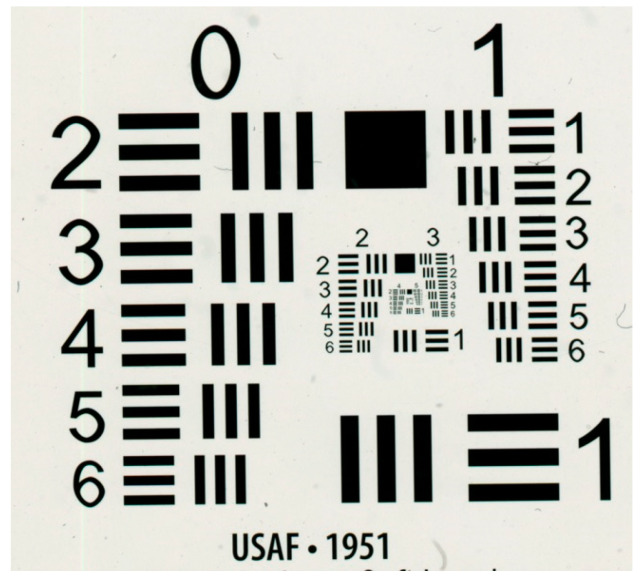
USAF 1951 resolution test chart.

In this experiment, we compared the USAF 1951 target at 150 Kelvin and 180 Kelvin. The USAF 1951 resolution test chart is shown in Figure 4. The test chart will be able to help determine the maximum optical resolution that we can obtain. As we can see, there are six groups. Each group consists of six elements, numbered from 1 to 6. The optical resolution is calculated as follows:

(9) $$resolution\left(\frac{lp}{mm}\right)=2^{group+(element-1)\times 6}$$

**Figure 5:**
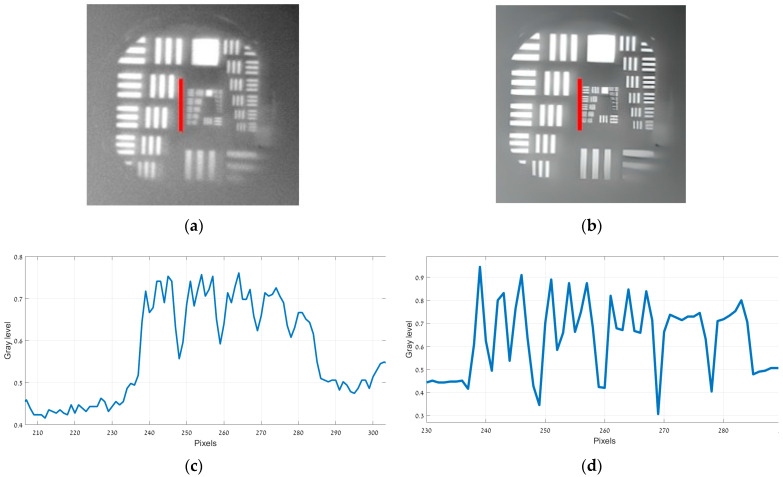
USAF 1951 images captured at 150 Kelvin. (**a**) Original image; (**b**) image after ESRGAN enhancement; (**c**) intensity profile of original image; (**d**) intensity profile after ESRGAN enhancement.

**Figure 6:**
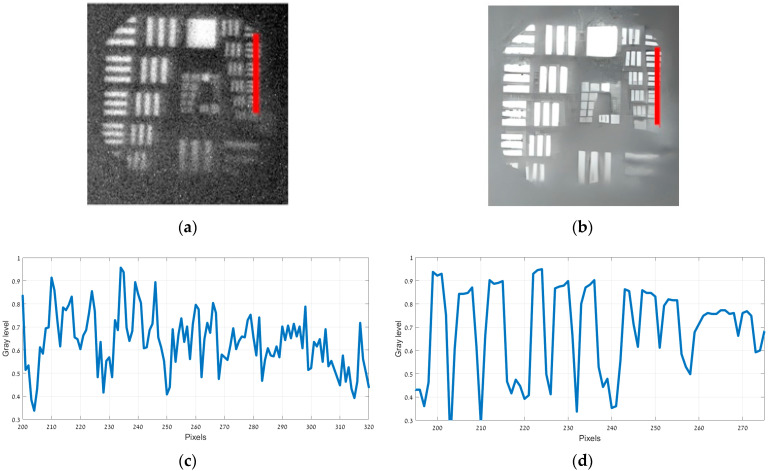
USAF 1951 images captured at 180 Kelvin. (**a**) Original image; (**b**) image after ESRGAN enhancement; (**c**) intensity profile of original image; (**d**) intensity profile after ESRGAN enhancement.

Figure 5a presents the original 150 Kelvin image, while Figure 5b displays its enhanced counterpart. The intensity profiles along the finest bar region (marked in red in both Figure 5a,b) are illustrated in Figure 5c,d. Figure 6a presents the original 180 Kelvin image, while Figure 6b displays its enhanced counterpart. The intensity profiles along the finest bar region (marked in red in both Figure 6a,b) are illustrated in Figure 6c,d.

Observing Figure 5b,d (150 Kelvin), the strips in group 2 element 4 are still discernible, indicating a resolution of 5.66 lp/mm. In contrast, Figure 6b,d (180 Kelvin) only shows detail up to group (−1) element 5, yielding a resolution of 0.794 lp/mm. In both temperature conditions, the enhanced images demonstrate an improvement over the original.

### 3.2. Second Experiment

**Figure 7:**
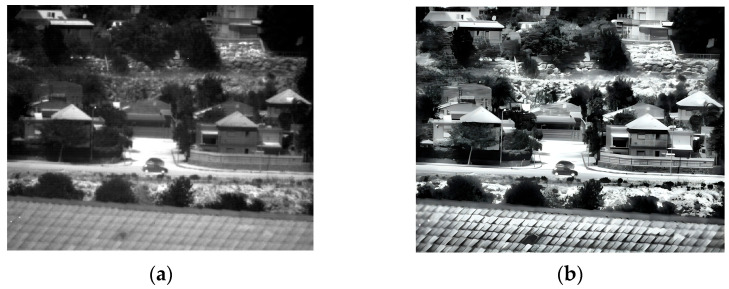
Daylight images captured at 150 Kelvin. (**a**) Original image; (**b**) after enhancement.

**Figure 8:**
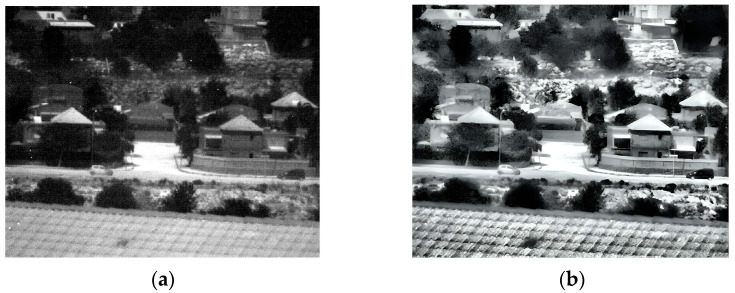
Daylight images captured at 180 Kelvin. (**a**) Original image; (**b**) after enhancement.

The second experiment was conducted outdoors during daylight hours. The pictures contain buildings, trees, and vehicles. The original image that was captured at 150 Kelvin and its enhancement are shown in Figure 7 and the 180 Kelvin original and enhancement images are shown in Figure 8. The original image in 180 Kelvin is blurred and it is hard to identify details such as the shape of the wall near the black car, the shape of the tree trunk near the white car, or even the fence in the upper right side of the image, which can be seen in the enhanced image. It is clear that the 150 Kelvin enhanced image is better quality compared with the 180 Kelvin image. However, the gap narrowed sharply after the enhancement.

### 3.3. Third Experiment

**Figure 9:**
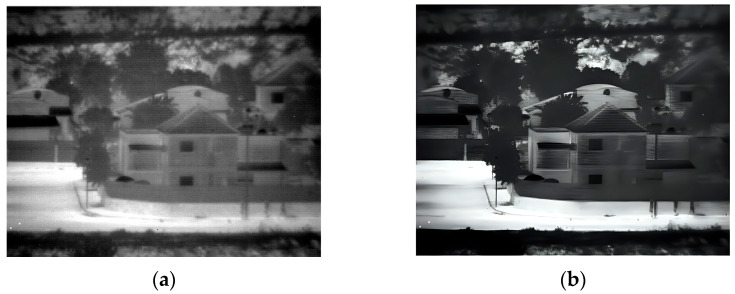
Low-light images captured at 150 Kelvin. (**a**) Original image; (**b**) after enhancement.

**Figure 10:**
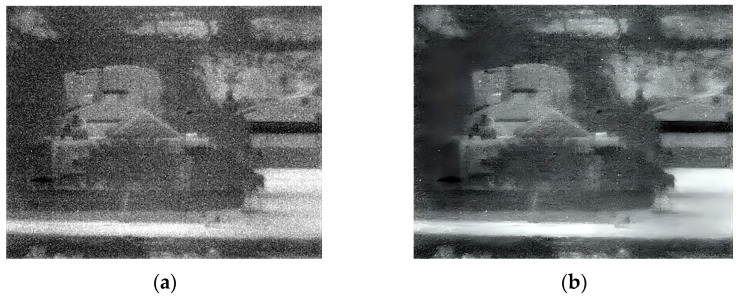
Low-light images captured at 180 Kelvin. (**a**) Original image; (**b**) after enhancement.

The third experiment took place outdoors in low-light evening conditions, capturing images of buildings, trees, and people. The original and enhanced 150 Kelvin images are shown in Figure 9, and the 180 Kelvin versions in Figure 10. Enhancement reduced blur and revealed more details in both temperature conditions, with the 150 Kelvin image exhibiting superior quality. For instance, Figure 9’s enhanced image clearly shows three people walking on the lower-right sidewalk, who were difficult to discern in the original. However, identifying the individual walking on the sidewalk in Figure 10 remains challenging even after enhancement.

### 3.4. Forth Experiment

**Figure 11:**
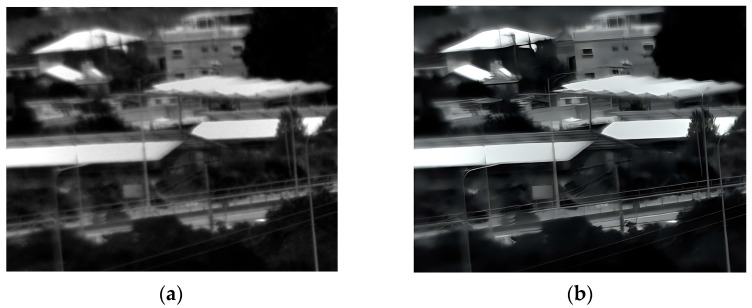
Daylight images (far distance) captured at 150 Kelvin. (**a**) Original image; (**b**) after enhancement.

**Figure 12:**
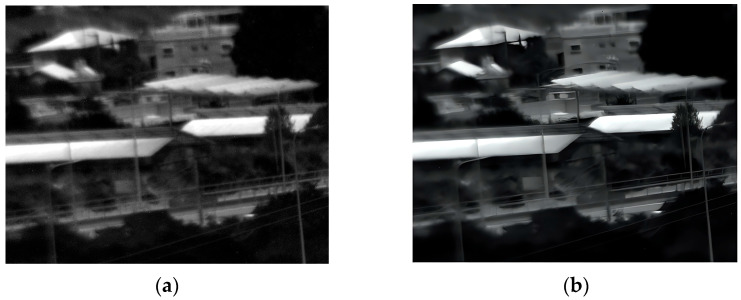
Daylight images (far distance) captured at 180 Kelvin. (**a**) Original image; (**b**) after enhancement.

Our fourth experiment involved capturing images of buildings and trees outdoors in daylight from a different distance than the second experiment. Figure 11 illustrates the original and enhanced 150 Kelvin images, and Figure 12 the 180 Kelvin counterparts. The enhancement process effectively reduced blur and revealed more detail at both temperatures, with a slight quality advantage observed in the 150 Kelvin result. However, because the scene lacked intricate details, the enhanced images at 150 Kelvin and 180 Kelvin appear substantially similar.

### 3.5. Fifth Experiment

**Figure 13:**
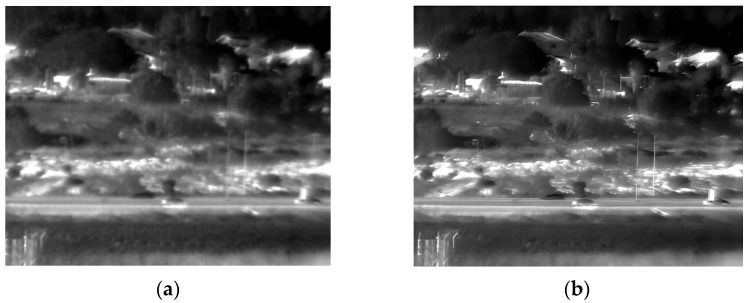
Open view images captured at 150 Kelvin. (**a**) Original image; (**b**) after enhancement.

**Figure 14:**
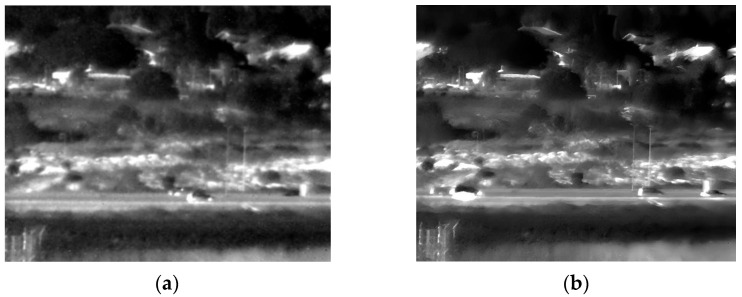
Open view images captured at 180 Kelvin. (**a**) Original image; (**b**) after enhancement.

The fifth experiment took place outdoors in open view conditions, capturing images of buildings, trees, and cars. The original and enhanced 150 Kelvin images are shown in Figure 13, and the 180 Kelvin versions in Figure 14. The enhancement process successfully reduced blur and revealed more details at both 150 Kelvin and 180 Kelvin, with the former showing better overall quality. For example, in the enhanced Figure 13, previously indistinct light poles on the road and even small windows in background village houses are now clearly visible. The 180 Kelvin enhanced image (Figure 14) also shows improved detail, although some elements like shrubs and trees remain slightly blurred.

To quantify the improvement, we made a comparison using the NIQE algorithm for the original images at both 150 and 180 Kelvin, as can be seen in Table 1 below. A lower score means that the images are considered to have higher perceived quality. One can see that image enhancement made a difference in almost all the images (except in the USAF 1951 images at 150 Kelvin, where the scores are quite the same in both images), and that the results for the enhanced 180 Kelvin images are similar to the enhanced 150 Kelvin images, although, the starting point of the 180 Kelvin images were worse.

Table 2 compares the original and enhanced images using MSE (where lower is better), UIQI (with 1 being a perfect match and 0 indicating no correlation), and PSNR (where higher scores, especially above 20, signify a good match). The results consistently show better metrics for the 150 Kelvin images compared with the 180 Kelvin images across all tested methods. This difference arises because the original 180 Kelvin images had lower initial quality, leading to larger improvements upon enhancement compared with the already-better quality 150 Kelvin original images. Interestingly, at longer distances where the focus shifts from small details to the overall scene, the enhanced 180 Kelvin images achieve a quality level similar to the enhanced 150 Kelvin images.

## 4. Discussion

The research presented in this article demonstrates the potential for operating xBn MWIR thermal cameras at 180 Kelvin. While the results are promising, especially for daylight conditions, there are several avenues for further exploration and improvement detailed in the following sections.

### 4.1. Algorithm Optimization

Algorithm optimization is crucial for enhancing the image quality of xBn MWIR thermal cameras operating at 180 Kelvin, especially under low-light conditions, and for reducing the computational cost of every run. There are several specific strategies for achieving this:

Training data specificity: The AI algorithm used in the study, ESRGAN, was not specifically trained for thermal images nor was the xBn MWIR camera being tested. Training deep learning models on datasets of thermal images captured at 180 Kelvin is essential. This allows the algorithm to learn the specific noise patterns and characteristics associated with higher temperature operation, leading to better noise reduction and image enhancement.

Exploring architectures: While ESRGAN has shown promise, there are other deep learning architectures for single-image super-resolution (SISR). Models like SRCNN, VDSR, EDSR, and SRGAN could be evaluated and compared based on their performance on 180 Kelvin thermal images. Each of these models has strengths and weaknesses, and selecting the optimal architecture will depend on factors such as the desired level of image detail, computational constraints, and the specific application.

Addressing low-light challenges: The sources emphasize the need for algorithms specifically designed to tackle low-light image enhancement. LIME (low-light image enhancement), which estimates and refines the illumination map of an image, could be particularly effective in this context. By addressing the uneven illumination often present in low-light scenes, LIME can improve the visibility of details and enhance overall image quality.

Computational cost: Optimizing the computational cost of a neural network often involves navigating a trade-off between resource efficiency and model quality. Techniques like network pruning, quantization, and knowledge distillation inherently aim to reduce computational demands (faster inference, smaller model size, and lower power consumption) but can potentially lead to a slight degradation in the model’s accuracy.

Incorporating attention mechanisms: Attention-based deep learning models, such as RCAN (Residual Channel Attention Network) [26], have shown significant potential for image super-resolution. These models selectively focus on specific features or regions of an image, allowing for more efficient processing and potentially leading to better results, particularly in challenging scenarios like low-light conditions.

By meticulously optimizing these algorithmic aspects, one can unlock the full potential of 180 Kelvin thermal imaging, leading to more robust, higher-quality images even in challenging lighting conditions. This advancement could revolutionize the field, making high-performance thermal imaging more accessible for various applications.

### 4.2. Multi-Image Processing

The current study focused on single image enhancement (SISO). By incorporating multi-image processing techniques, such as temporal noise reduction or super-resolution algorithms that leverage information from consecutive frames, it may be possible to achieve significant improvements in image quality, even in low-light scenarios. This approach could be particularly beneficial for capturing moving objects, as algorithms designed to identify differences between consecutive images could be employed. Additionally, exploring multi-spectral and spatial data fusion techniques, similar to those used in remote sensing, could further enhance resolution and image detail by combining information from multiple spectral bands.

By pursuing these research directions, the feasibility and effectiveness of 180 Kelvin thermal imaging can be further validated and enhanced, potentially paving the way for a new generation of cost-effective and high-performance thermal imaging systems across various applications.

## 5. Conclusions

This research demonstrates that operating xBn MWIR thermal cameras at 180 Kelvin is achievable, particularly in daylight conditions where high-quality images can be obtained even with generic image processing. However, there are still some challenges remaining in enhancing image quality in low-light scenarios. This study underscores the importance of continued research and development in enhancement algorithms, emphasizing the need for training on extensive thermal image datasets and optimization for LIME. By addressing these research areas, the feasibility and effectiveness of 180 Kelvin thermal imaging can be further enhanced. This advancement could lead to a new generation of cost-effective and high-performance thermal imaging systems for diverse applications, including astronomy, environmental monitoring, and materials science.

## Figures and Tables

**Table 1:** NIQE results to all images.

Original Image	Original 150 k	Enhanced 150 k	Original 180 k	Enhanced 180 k
USAF1951	3.637	3.76	12.421	5.73
Daylight	4.418	2.178	5.328	1.928
Low light (evening)	7.56	3.803	16.794	3.033
Daylight (far distance)	4.57	4.11	4.68	3.99
Open view	4.76	3.42	4.44	2.99

**Table 2:** MSE/PSNR/UIQI results for original/enhanced pair images comparison.

Pair Images	MSE	PSNR	UIQI
USAF1951 150 original/150 enhanced	0.013	18.846	0.826
USAF1951 180 original/180 enhanced	0.004	23.947	0.954
Daylight 150 original/150 enhanced	0.036	14.041	0.787
Daylight 180 original/180 enhanced	0.045	13.44	0.751
Low light (evening) 150 original/150 enhanced	0.014	18.42	0.902
Low light (evening) 180 original/180 enhanced	0.019	17.05	0.775
Daylight (far distance) 150 original/150 enhanced	0.002	28.02	0.987
Daylight (far distance) 180 original/180 enhanced	0.001	30.14	0.99
Open view 150 original/150 enhanced	0.007	21.64	0.925
Open view 180 original/180 enhanced	0.003	24.89	0.97

## Data Availability

The raw data supporting the conclusions of this article will be made available by the authors on request.
